# T cells in mesenteric and subcutaneous adipose tissue of Holstein-Friesian cows

**DOI:** 10.1038/s41598-019-39938-0

**Published:** 2019-03-04

**Authors:** Bárbara M. Oliveira, Ana M. Rasteiro, Alexandra Correia, Ana Pinto, Pedro Meireles, Paula G. Ferreira, Manuel Vilanova, Luzia Teixeira

**Affiliations:** 1 ICBAS – Instituto de Ciências Biomédicas Abel Salazar, Universidade do Porto, Rua de Jorge Viterbo Ferreira, 228, 4050-313 Porto, Portugal; 2 UMIB –Unidade Multidisciplinar de Investigação Biomédica, Universidade do Porto, Rua de Jorge Viterbo Ferreira, 228, 4050-313 Porto, Portugal; 3 I3S-Instituto de Investigação e Inovação em Saúde, Universidade do Porto, Rua Alfredo Allen, 208, 4200-135 Porto, Portugal; 4 IBMC – Instituto de Biologia Molecular e Celular, Rua Alfredo Allen, 208, 4200-135 Porto, Portugal; 5 SVAExpleite, Rua D. Sancho I, 3202, 4760-485 Vila Nova de Famalicão, Portugal

## Abstract

The importance of immune cells present in the adipose tissue to metabolic homeostasis has been increasingly recognized. Nevertheless, in bovines few studies have so far addressed the immune cell populations resident in this tissue. Here we developed an eight-colour flow cytometry panel to address T cell populations present in bovine adipose tissue. Our results showed that γδ T cells, CD4^+^ and CD8^+^ CD3^+^ non-γδ T cells, as well as NK cells, are present in the mesenteric and subcutaneous adipose tissue of Holstein-Friesian cows. The frequency of both γδ T cells and CD8^+^ non-γδ T cells was found higher in mesenteric than in subcutaneous adipose tissue. The majority of T cells in adipose tissue presented a CD45RO^+^CD62L^−^ phenotype, characteristic of effector memory cells, and the frequency of these cellular populations was higher than in the blood. The ratio of CD4^+^ T cells over CD8^+^ T cells was similar between subcutaneous and mesenteric adipose tissue but different from the one found in blood. Overall, our results highlight particular phenotypic characteristics of bovine adipose tissue T cell populations.

## Introduction

The adipose tissue is no longer regarded as a simple organ for lipid storage, but is recognized as an endocrine organ important for metabolism homeostasis^1,2^. It has been shown that immune cell populations resident in adipose tissue have a critical role in regulation of not only local but also systemic metabolic homeostasis^3,4^. Studies in humans and mice have shown that cells like γδ and NK T cells are enriched in the adipose tissue and have an important role in maintaining its homeostasis^3,5^. These populations interact with and promote T regulatory cells (Treg), an important population for the maintenance of an anti-inflammatory environment in that tissue^3,5^. On the other hand, an increase in CD8^+^ T cells has been associated with inflammation in the adipose tissue^6^. Despite the acknowledged importance of these cellular populations in murine and human adipose tissue, little is known regarding lymphocyte populations present in the adipose tissue of bovines. Contreras *et al*. reported the presence of CD3^+^ cells and B cells in the stromal vascular fraction of subcutaneous and omental adipose tissue^7,8^. However, T cell subpopulations in those tissues were not characterized. Moreover, recent studies in mice and humans have shown that the adipose tissue is a reservoir for memory T cells with potential to protect from infection^9^. CD45RO is a marker of CD4^+^ and CD8^+^ memory T cells in humans^10,11^ that has also been used to identify bovine memory CD4^+^ T cells^12^. Studies with *Theileria parva* showed that memory CD8^+^ T cells (assessed by cytotoxic capacity) were found in both CD45RO^+^ and CD45RO^−^ subsets^12^. More recently, others have shown that memory responses (assessed by IFN-γ production and cytotoxic capacity) to mycobacteria were due to CD45RO^+^ but not to CD45RO^−^ CD8^+^ T cells^13^. It has also been shown that CD45RO^+^CD62L^+^ and CD45RO^+^CD62L^−^ CD4^+^ T cells contribute more to IFN-γ production in BCG vaccinated and *Mycobacterium bovis* infected cattle than CD45RO^−^CD62L^+^ CD4^+^ T cells^14^. CD62L can be found in both memory and naïve T cells^15^. In bovines, pathogen-specific CD4^+^ T cells with central memory characteristics were shown to express CD62L^16^. In a very simplified classification, as suggested by others, effector memory, central memory and naïve cells would respectively display the CD45RO^+^CD62L^lo^, CD45RO^+^CD62L^hi^ and CD45RO^−^CD62L^lo/hi^ surface phenotypes^14^. Therefore, in this work, we aimed at characterizing lymphocyte cell subpopulations present in the bovine adipose tissue, assessing the presence of memory T cell subpopulations therein by using a combination of CD45RO and CD62L markers. The obtained results altogether show that in the bovine adipose tissue T cells predominate with a memory-like phenotype.

## Results

### Flow cytometry panel

**Figure 1:**
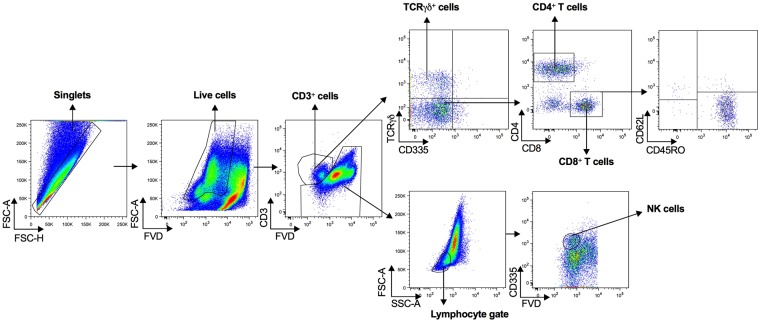
Lymphocyte subpopulations. Flow cytometry gating strategy used to define γδ T cells (TCRγδ^+^CD3^+^CD335^−^), CD4^+^ T cells (CD4^+^CD3^+^TCRγδ^−^CD335^−^), CD8^+^ T cells (CD8^+^CD3^+^TCRγδ^−^CD335^−^) and NK cells (CD335^+^CD3^−^) in the stromal vascular fraction (SVF) of mesenteric and subcutaneous bovine adipose tissue (MAT and SAT, respectively) and in peripheral blood leukocytes. Dead cells were excluded with Fixable Viability Dye (FVD), lymphocytes were gated based on SSC-A versus FSC-A and singlets were selected from the FSC-A versus FSC-H dot plot. The flow cytometry gating strategy used to define CD45RO^+^ and CD62L^+^ T cell subpopulations is also shown in CD8^+^ T cells. Pseudocolor plots are representative examples with SVF cells isolated from MAT.

T lymphocytes subpopulations were assessed in bovine adipose tissue from different anatomical locations, namely: mesenteric adipose tissue (MAT) and subcutaneous adipose tissue (SAT) using an eight-colour flow cytometry panel (Fig. 1). For comparison, peripheral blood leukocytes (PBL) were isolated and similarly stained. A fixable viability dye (FVD) was included in our panel to exclude interference from dead cells. NK cells were selected using the CD335 (NKp46) cell surface marker^17^. Although this marker was initially reported to be present only in NK cells^17^, it was reported to be also expressed in a population of CD3^+^ cells in cattle^18^. Therefore, NK cells were selected from the CD3-negative population. In the CD3^+^ population, γδ T cells were selected by using an antibody specific to the δ chain^19^. Only CD335^−^ cells were included in the analysis. CD4^+^ and CD8^+^ T cells were selected from the CD3^+^ TCRγδ^−^ CD335^−^ population. After selection of the different populations, CD45RO and CD62L markers were used to define CD45RO^+^CD62L^−^, CD45RO^+^CD62L^+^, CD62L^+^CD45RO^−^ and CD45RO^−^CD62L^−^ populations. In Supplementary Fig. S1, examples of fluorescence minus one (FMO) controls, used to define the gates shown in Fig. 1, are given for MAT, SAT and PBL for the markers that were more prone to gate selection error.

### γδ T cells

**Figure 2:**
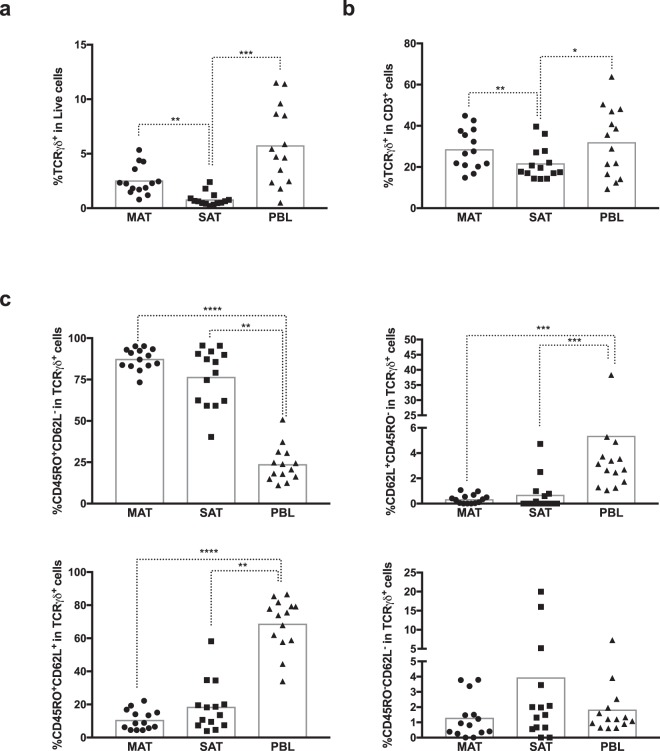
Expression of memory markers in γδ T cells. Frequencies of TCRγδ^+^ cells (TCRγδ^+^CD3^+^CD335^−^) in (**a**) total live stromal vascular fraction cells and (**b**) CD3^+^ cells isolated from mesenteric and subcutaneous bovine adipose tissue (MAT and SAT, respectively) and in peripheral blood leukocytes (PBL). (**c**) Frequencies of CD45RO^+^CD62L^−^, CD62L^+^CD45RO^−^, CD45RO^+^CD62L^+^ and CD45RO^−^CD62L^−^ cells on total TCRγδ^+^ cells in MAT, SAT and PBL. Each symbol represents an individual animal. Bars represent means of 14 bovines per group pooled from 5 independent experiments. Statistically significant differences between different tissues are indicated. (Friedman test with Dunn’s multiple comparisons test; *P < 0.05; **P < 0.01; ***P < 0.001; ****P < 0.0001).

γδ T cells are found in adipose tissue of mice and humans^5,20–22^. Here we show by flow cytometry analysis (Fig. 2) and immunocytochemistry (Supplementary Fig. S2) that γδ T cells are also present in the stromal vascular fraction (SVF) of the bovine adipose tissue. The frequency of γδ T cells among total SVF cells was found to be higher in MAT than in SAT (Fig. 2a). The latter also presented lower proportions of γδ T cells than the ones detected in PBL. When we analysed the frequency of γδ T cells in total CD3 cells, the same differences were observed (Fig. 2b): higher frequency in MAT comparatively to SAT and higher frequency in PBL when compared to SAT. No differences in γδ T cell frequencies were observed between MAT and PBL. γδ T cells accounted for 14,8 to 44,9% of total CD3 cells in MAT and 14,2 to 39,6% in SAT. In peripheral blood the frequency in CD3 population ranged from 9,31 to 63,8%. Cells expressing both CD335 and TCRγδ were detected in PBL (Supplementary Fig. S1). As the number of events obtained for this cellular population in the flow cytometry analysis was very reduced in adipose tissue samples, and the gate region was not always easy to define (Supplementary Fig. S1), we did not analyse this region. Memory-like γδ T cell responses have been described not only in humans and mice^23^ but also in bovines^24^. The majority of γδ T cells express the memory marker CD45RO both in adipose tissue and PBL (Fig. 2c and Supplementary Figs S3–5). In the adipose tissue the frequency of CD45RO^+^CD62L^−^ cells was higher than in PBL, whereas the inverse was observed regarding the CD45RO^+^CD62L^+^ and CD62L^+^CD45RO^−^ phenotypes (Fig. 2c). Indeed, the number of events for CD62L^+^CD45RO^−^ population in the adipose tissue was very scarce or even undetected in some animals (not detected in three animals in MAT and eight animals in SAT). The CD45RO^+^CD62L^−^ phenotype was the most frequent within adipose tissue γδ T cells, except for one animal in SAT (Supplementary Figs S3–4). Contrastingly, in the blood, the majority of γδ T cells presented a CD45RO^+^CD62L^+^ phenotype, except for two animals where CD62L^+^CD45RO^−^ and CD45RO^+^CD62L^−^ phenotypes were the most frequent (Supplementary Fig. S5).

### CD4^+^ and CD8^+^ T cells

**Figure 3:**
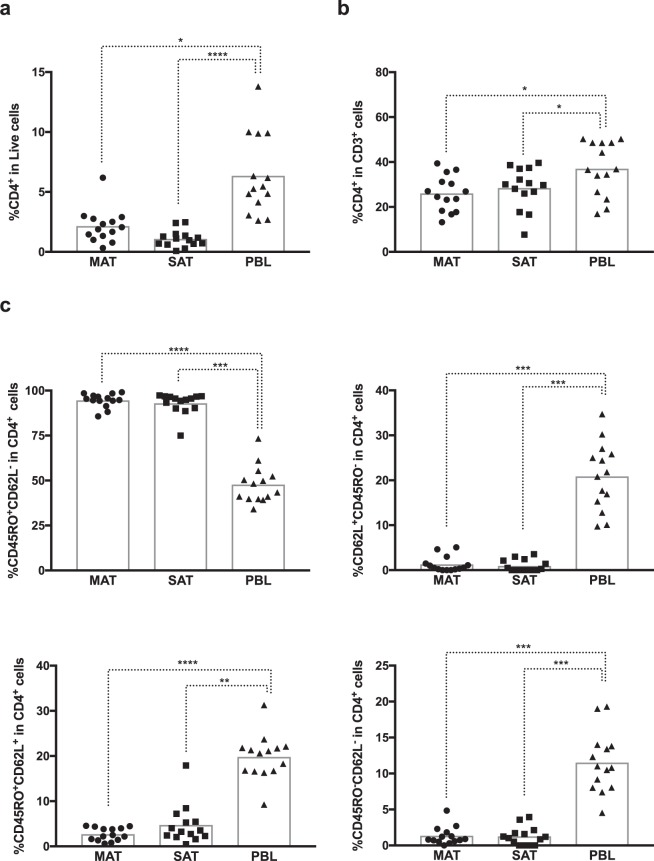
Expression of memory markers in CD4^+^ T cells. Frequencies of CD4^+^ T cells (CD4^+^CD3^+^TCRγδ^−^CD335^−^) in (**a**) total live stromal vascular fraction cells and (**b**) CD3^+^ cells isolated from mesenteric and subcutaneous bovine adipose tissue (MAT and SAT, respectively) and in peripheral blood leukocytes (PBL). (**c**) Frequencies of CD45RO^+^CD62L^−^, CD62L^+^CD45RO^−^, CD45RO^+^CD62L^+^ and CD45RO^−^CD62L^−^ cells on total CD4^+^ T cells in MAT, SAT and PBL. Each symbol represents an individual animal. Bars represent means of 14 bovines per group pooled from 5 independent experiments. Statistically significant differences between different tissues are indicated. (Friedman test with Dunn’s multiple comparisons test; *P < 0.05; **P < 0.01; ***P < 0.001; ****P < 0.0001).

As no antibody targeting the bovine TCR α or β chains^25^ was currently available, we defined the αβ T cell population as CD3^+^TCRγδ^−^ (Fig. 1). CD3^+^TCRγδ^−^ cells expressing CD335 were detected in blood and adipose tissue (Fig. 1 and Supplementary Fig. S1). As few events were detected in the adipose tissue, we did not analyse this cellular population any further. CD4^+^ T cells were present at lower frequencies in adipose tissue total SVF cells or CD3^+^ cells than in PBL (Fig. 3a,b). In MAT and SAT, CD4^+^ T cells accounted for 13,2 to 39,4% and 7,7 to 39,6% of total CD3^+^ T cells, respectively, while in PBL they ranged from 16,9 to 50,3%.

In the adipose tissue the frequency of CD45RO^+^CD62L^−^ cells within CD4^+^ T cells was higher than in PBL, whereas the inverse was observed for the CD45RO^+^CD62L^+^ and CD62L^+^CD45RO^−^ phenotype (Fig. 3c). As for γδ T cells, CD4^+^CD3^+^TCRγδ^−^ cells with a CD62L^+^CD45RO^−^ phenotype were very scarce in adipose tissue, and were not detected in three animals in MAT and seven animals in SAT. The CD45RO^+^CD62L^−^ phenotype was the one predominating in both the adipose tissue and blood in all animals analysed (Supplementary Figs S6–8).

**Figure 4:**
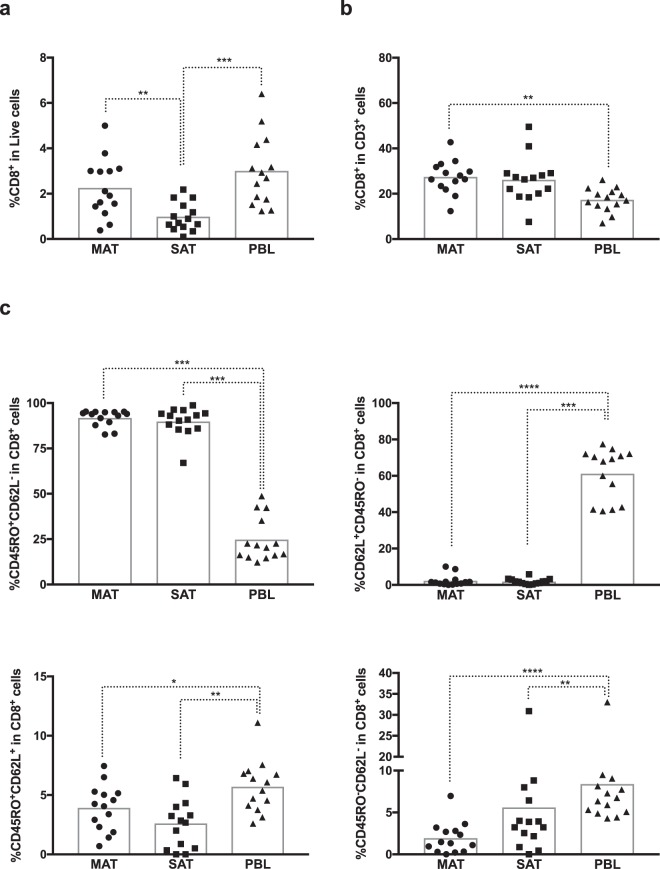
Expression of memory markers in CD8^+^ T cells. Frequencies of CD8^+^ T cells (CD8^+^CD3^+^TCRγδ^−^CD335^−^), in **(a)** total live stromal vascular fraction cells and **(b)** CD3^+^ cells isolated from mesenteric and subcutaneous bovine adipose tissue (MAT and SAT, respectively) and in peripheral blood leukocytes (PBL). **(c)** Frequencies of CD45RO^+^CD62L^−^, CD62L^+^CD45RO^−^, CD45RO^+^CD62L^+^ and CD45RO^−^CD62L^−^ cells on total CD8^+^ T cells in MAT, SAT and PBL. Each symbol represents an individual animal. Bars represent means of 14 bovines per group pooled from 5 independent experiments. Statistically significant differences between different tissues are indicated. (Friedman test with Dunn’s multiple comparisons test; *P < 0.05; **P < 0.01; ***P < 0.001; ****P < 0.0001).

In mice, CD8^+^ T cells account for 3–5% of all SVF cells^26^. In the analysed bovines, the frequency of these cells within total SVF cells ranged from 0,386 to 3,78% in MAT and 0,105 to 2,18% in SAT. The CD8^+^ T cell population is at higher frequency in MAT than in SAT, and at a higher frequency in PBL comparatively to SAT (Fig. 4a). Contrastingly, when we analysed the frequency of CD8^+^ T cells within CD3^+^ T cells, no difference was found between MAT and SAT (Fig. 4b). A higher frequency of CD8^+^ T cells was observed in MAT comparatively to PBL. CD8^+^ T cells accounted for 12,3 to 42,7% of total CD3^+^ T cells in MAT; 7,6 to 49,5% in SAT and 6,99 to 26,1% in PBL. As observed for CD4^+^ T cells, the frequency of CD8-expressing CD45RO^+^CD62L^−^ T cells in adipose tissue was higher than in PBL, whereas the inverse was observed for cells with a CD45RO^+^CD62L^+^ and CD62L^+^CD45RO^−^ phenotype (Fig. 4c). However, while in adipose tissue (MAT and SAT) the majority of CD8^+^ T cells presented a CD45RO^+^CD62L^−^ phenotype in all animals analysed, in blood the most frequent phenotype, in all but two animals, was CD62L^+^CD45RO^−^ (Supplementary Figs S9–11). The CD4^+^: CD8^+^ T cell ratio was found approximately two-fold higher in PBL comparatively to adipose tissue (Supplementary Fig. S12).

### NK cells

**Figure 5:**
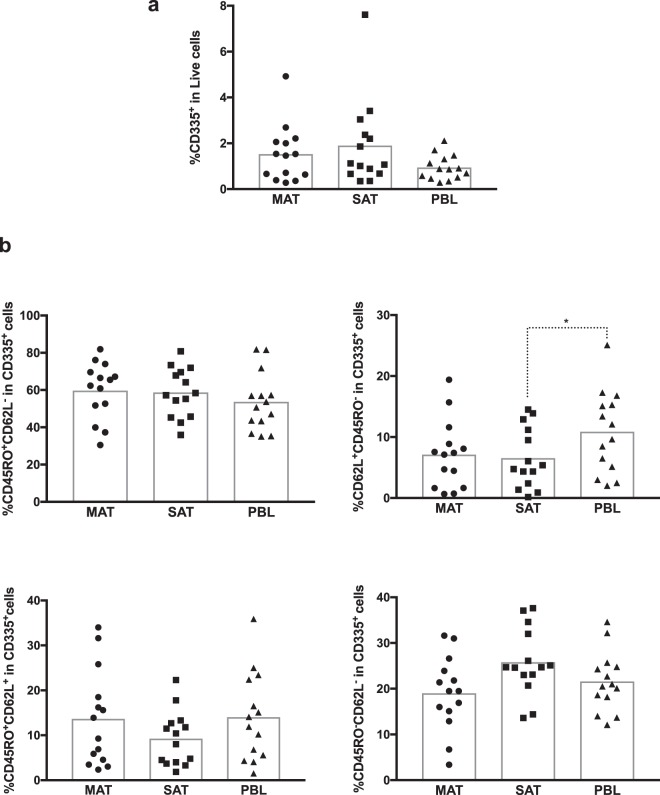
NK cells in bovine adipose tissue. (**a**) Frequencies of NK cells (CD335^+^CD3^−^), in total live stromal vascular fraction cells isolated from mesenteric and subcutaneous bovine adipose tissue (MAT and SAT, respectively) and in peripheral blood leukocytes (PBL). (**b**) Frequencies of CD45RO^+^CD62L^−^, CD62L^+^CD45RO^−^, CD45RO^+^CD62L^+^ and CD45RO^−^CD62L^−^ cells on total CD335^+^CD3^−^ in MAT, SAT and PBL. Each symbol represents an individual animal. Bars represent means of 14 bovines per group pooled from 5 independent experiments. Statistically significant differences between different tissues are indicated. (Friedman test with Dunn’s multiple comparisons test; *P < 0.05).

NK cells were previously described in adipose tissue of mice and humans^27,28^. We show here the presence of NK cells in the bovine adipose tissue SVF at frequencies similar to those in PBL (Fig. 5a). Regarding the markers CD45RO and CD62L no major difference was observed between the adipose tissue and PBL, exception for a slightly higher frequency of CD62L^+^CD45RO^−^ within CD335^+^ cells in PBL comparatively to SAT. In both MAT and SAT, the CD45RO^+^ CD62L^−^ phenotype was predominant among CD335^+^ cells in all 14 animals (Fig. 5b and Supplementary Figs S13–14). The same holds true for PBL except in one animal (Supplementary Fig. S15).

## Discussion

Here we constructed an 8-colour panel for the analysis of T lymphocytes as it allowed a more thorough characterization of the different subsets present in the adipose tissue. T cells expressing the γδ antigen receptor represent a major circulating lymphocyte subtype in cattle, especially in young animals where they can account up to 60% of total T cells in blood^29^. Here we show that γδ T cells are also abundant in bovine adipose tissue representing up to 45% of all T cells in MAT and up to 40% in SAT. Others have shown that in male mice γδ T cells are more abundant in adipose tissue than in blood^5^. In humans, higher frequency of γδ T cells in total CD45^+^ cells was observed in omentum comparatively to peripheral blood^5^. Contrastingly, in bovine adipose tissue we do not observe higher frequencies of γδ T cells comparatively to blood. As already described by others, in bovine blood the majority of γδ T cells are CD45RO positive^12^. We showed here that in the adipose tissue nearly all γδ T cells are also CD45RO^+^. However, contrastingly to blood, the majority of these cells do not co-express CD62L. Similarly, others have already shown in bovines that while the majority of WC1^+^ γδ T cells in the blood express CD62L, they were mainly CD62L negative in the gut mucosa^15^. In mouse epididymal adipose tissue 75% of γδ T cells are CD44^high^ CD62L^low 22^. It has been recognized that γδ T cells can participate in immunological memory^23^. It would therefore be interesting in future studies to assess whether this phenotype could result from specific antigen stimulation or to local cues provided by the adipose tissue environment.

We also observed that the majority of TCRγδ^−^ CD4^+^ and CD8^+^ T cells in the bovine adipose tissue were CD62L negative. This phenotype is also displayed in the majority of CD4^+^ and CD8^+^ T cells resident in the gut mucosa that contrasts to the ones in blood where a large fraction was CD62L^+^^15^. Others have shown that in the lungs, the majority of CD8^+^ T cells express CD45RO (94,81%), with only 6.794% of CD8^+^ T cells expressing CD62L^30^. The prevailing CD45RO^+^CD62L^−^ phenotype found in bovine adipose tissue T cells is characteristic of memory T cells. These can be effector or tissue resident memory cells as those present in mouse adipose tissue^9^, that cannot be discriminated by the markers used^31^. While in the blood the majority of CD4^+^ T cells exhibited the CD45RO^+^CD62L^−^ phenotype, as also reported by others^14^, CD8^+^ T cells majorly display a CD62L^+^CD45RO^−^ naïve phenotype. In humans a phenotypic difference was also observed between CD4^+^ and CD8^+^ peripheral blood T cells^11^. Interestingly, the CD4^+^:CD8^+^ T cell ratio was higher in the PBL than in the adipose tissue. This may indicate a preferential recruitment/retention of CD8^+^ T cells into the adipose tissue. Alternatively, whether these two lymphocyte populations may have a different cell turnover in the adipose tissue environment would also be interesting to assess.

In the CD3^−^ population we observed a population expressing CD335 that we define as NK cells. The slight variation observed in the frequency of NK cells can be due to the random selection of the animals. The percentage of this cellular population as well as that of TCRγδ cells in peripheral blood can be influenced by age^32,33^, which can be a possible explanation for some of the variability observed in our data. As reported by others^33^ we observed that a large proportion of NK cells in blood express the marker CD45RO. In bovines, CD45RO expression in NK cells can be regulated upon IL-2 stimulation^34^. Studies in humans have shown that CD45RO^+^ NK cells from pleural fluid cells produce more IFN-γ than CD45RO^−^ NK cells in response to IL-12^35^. Nevertheless, contrastingly to what was observed for T cells, no differences were found in the frequency of cells expressing CD45RO in adipose tissue comparatively to blood. The lymph node homing receptor CD62L was also shown to be expressed by NK cells in the blood^36^, an observation we confirm here.

Overall, we show here that several lymphocyte subpopulations exist in bovine adipose tissue phenotypically distinct from the ones found in blood. Namely, in adipose tissue the majority of γδ, CD4^+^ and CD8^+^ T cells have an effector memory-like phenotype, CD45RO^+^CD62L^−^. Our results also showed that SVF cell populations presented different proportions in adipose tissue from different anatomical locations. Namely, higher frequencies of γδ T cells and CD8^+^ T cells were observed in MAT when compared to SAT. It is known that diverse factors can influence the number of cells in the adipose tissue. Previous studies have shown that circulating CD8^+^ T and NK cells accumulate in the visceral adipose tissue (VAT) of mice fed a high fat diet (HFD) compared to normal diet control^6,27^. Studies in bovines have shown increased frequencies of cells expressing CD14 and CD172a in omental adipose and CD11c and CD163 in omental and subcutaneous adipose tissue of cows with displaced abomasum^7^. Increased numbers of CD172a^+^ cells were also observed in omental and subcutaneous adipose tissue upon feed restriction^8^. It is also known from studies done in the murine model that infection can lead to significant and long-lasting alterations in the frequencies of lymphocyte cell populations in the adipose tissue^37–39^.

Although the animals analysed here have been randomly selected at the slaughterhouse, a clear distinct frequency of T cell subtypes in adipose tissue comparatively to blood was observed. Moreover, our results also show a distinct compartmentalization of CD4^+^ and CD8^+^ T cells expressing the CD45RO and CD62L markers, that was consistently observed in the different animals, with predominance of effector memory cells in the adipose tissue. The specificity of these T cell populations resident in adipose tissue is however unknown. Whether antigen-dependent stimulation may induce this phenotype would be worth assessing in future studies.

## Methods

### Animals

Samples were removed from 14 female Holstein-Friesian cattle (*Bos taurus*) at a local slaughterhouse. Detailed information of age for all animals included in this study is presented in Supplementary Table 1. Tissue samples were randomly obtained from animals slaughtered for human consumption and therefore no animals were sacrificed for research purpose. Authorization to use animal’s by-products was given by Direção Geral de Alimentação e Veterinária (0421/000/000/2015) and Institutional Ethical Committee.

### Sample Collection

Samples of peripheral blood, mesenteric adipose tissue (MAT) and subcutaneous adipose tissue (SAT) were collected right after slaughter. Blood samples were collected from the jugular vein into tubes with EDTA (ethylene diamine tetracetate, BD Vacutainer^®^). SAT (removed from the flank region) and MAT (collected from the fat surrounding the mesenteric lymph nodes, avoiding the lymph nodes) were placed in Dulbecco’s Modified Eagle Medium (DMEM) supplemented with 100 units/mL penicillin, 100 μg/mL streptomycin, 250 ng/mL amphotericin B and 10 mM HEPES buffer (all from Sigma-Aldrich, St Louis, USA) and transported immediately to the laboratory for further analysis in a warm container.

### Isolation of peripheral blood leukocytes

For peripheral blood leukocytes (PBL) isolation, whole blood was incubated with red blood lysis buffer solution [162,64 mM NH_4_Cl (Sigma-Aldrich), 9.98 mM Tris base (Merck), pH = 7,2]. Cells were passed through a 100-μm cell strainer, washed and resuspended in Dulbecco’s PBS, supplemented with 2% FBS (Gibco, MA, USA), 2 mM EDTA and 10 mM HEPES (all from Sigma-Aldrich) after centrifugation at 300 g for 5 min at 4 °C.

### Isolation of stromal vascular fraction cells

Stromal vascular fraction (SVF) cells were isolated by a previously described methodology, with slight modifications^20,37^. Briefly, small pieces of adipose tissue (avoiding blood vessels) were added to tubes containing Hanks’ balanced salt solution supplemented with 4% BSA, 10 mM HEPES and Liberase™ TL Research Grade (Roche Diagnostics, Risch-Rotkreuz, Switzerland). After water bath incubation, digested samples were homogenized to single-cell suspensions, passed through a 100-μm cell strainer (BD Biosciences Pharmingen, San Diego, CA) and centrifuged at 280 × g for 10 min at 4 °C. Cells at the bottom, corresponding to the SVF were resuspended in Dulbecco’s PBS, supplemented with 2% FBS (Gibco, MA, USA), 2 mM EDTA and 10 mM HEPES (all from Sigma-Aldrich).

### Antibodies

As commercially available antibodies for bovines are conjugated to a very limited number of fluorochromes, to construct an eight-colour multicolour panel, some of the antibodies were first labelled with conjugation kits accordingly to manufacture instructions. Namely, mouse anti-bovine TCRγδ (Clone GB21A, Washington State University, Pullman, WA) was conjugated to Fluorescein isothiocyanate (FITC) with FITC conjugation kit (Bio-Rad, Kidlington, UK), mouse anti-bovine CD335 (clone AKS1, Bio-Rad) was conjugated to peridinin-chlorophyll protein-cychrome 5.5 (PerCP-Cy5.5) with PerCP-Cy5.5 conjugation kit (PerCP-Cy5.5) (Bio-Rad), mouse anti-bovine CD45RO (clone IL-A116, Bio-Rad) was conjugated to PE-cychrome 7 (PE-Cy7) with PE-Cy7^®^ conjugation kit (Abcam, Cambridge, UK), mouse anti-Bovine CD62L (clone BAQ92A, Washington State University) was conjugated to allophycocyanin Cyanine 7 (APC-Cy7) with APC-Cy7^®^ conjugation kit (Abcam) and mouse anti-bovine CD3 (Clone MM1A, Washington State University) was conjugated to CF™405 M with Mix-n-Stain™CF™405 M Antibody Labeling Kits (Sigma-Aldrich). Phycoerythrin (PE) anti-bovine CD8α (clone CC63) and Alexa Fluor 647^®^ anti-bovine CD4 (clone CC8) (all Bio-Rad) were commercially available. All antibodies were titrated and optimal concentration for adipose tissue samples determined.

### Flow cytometric analysis

For dead cell exclusion all samples, except single-stained and Fluorescence minus one (FMO) controls, were first incubated with eFluor^®^ 506 Fixable Viability Dye (eBioscience, San Diego, CA) diluted 1: 1000 in Dulbecco’s PBS for 30 min at 4 °C. Cells were then washed 2 times with PBS. Before surface staining, cells were incubated with 100 μg/mL of purified bovine IgG (Sigma-Aldrich) in Dulbecco’s PBS, 2% FBS, 2 mM EDTA, 10 mM HEPES as a blocking reagent for elimination of nonspecific binding, similarly to what is done for human studies^40^. SVF cells were then surface stained for 30 min with the above-described monoclonal antibodies. Briefly, FITC anti-bovine TCRγδ, PE anti-bovine CD8α, PerCP-Cy5.5 anti-bovine CD335, PE-Cy7 anti-bovine CD45RO, Alexa Fluor 647^®^ anti-bovine CD4, APC-Cy7 anti-bovine CD62L and CF405M- anti-bovine CD3. Following primary antibody incubation, cells were washed, fixed with 2% formaldehyde, washed and resuspended in Dulbecco’s PBS, 2% FBS, 2 mM EDTA, 10 mM HEPES. In each experiment for gating selection, FMO controls were made for all markers used using at least one adipose tissue sample. Whenever possible, FMO controls were made with one sample of MAT and one sample of SAT, at least for the markers that were more prone to gate selection error, namely for the markers TCRγδ, CD335, CD62L and CD3. FMO controls with PBL were also included for TCRγδ, CD335, CD45RO, CD62L and CD3 markers. Isotype controls were not used for gate setting as they have been shown to be highly unreliable^40^. 5 × 10^5^ to 1 × 10^6^ total cells were stained. Adipose tissue: 3 × 10^4^–6 × 10^5^ total events were acquired per sample, from which 1 × 10^4^–1 × 10^5^ cells, gated within live cells, were analysed. Peripheral blood: 4 × 10^5^ to 8 × 10^5^ total events were acquired per sample, from which 2 × 10^5^ to 7 × 10^5^ cells, gated within live cells, were analysed. Data acquisition was performed in a FACSCanto^TM^ II system (BD Biosciences, San Jose, CA) using the FACSDIVA^TM^ software (BD) and compensated and analysed in FlowJo version 9.9.6. (FlowJo LLC, Ashland, OR). Beads (antibodies) or cell (FVD) staining were used for compensation. The results are presented as the frequency of cells within live cells or frequency within each cellular population. A biexponential transformation was applied to improve data visualization.

### Immunocytochemistry

Cytospins of SVF cells isolated from MAT and SAT as well as leukocytes isolated from whole blood of dairy cattle were prepared using 1 to 3 × 10^5^ cells. The slides were then methanol fixed and stained for TCR γδ by a previously described protocol with some modifications^20^. Briefly, peroxidase activity was blocked by treatment with 3% hydrogen peroxide in methanol (Merck, Darmstadt, Germany) for 20 min. Slides were then incubated in a moist chamber for 20 min with normal rabbit serum (Dako, Glostrup, Denmark) diluted 1:5 in 10% BSA (Sigma), to eliminate non-specific staining. Excess serum was removed and the slides were incubated in a moist chamber overnight at 4 °C, with a monoclonal mouse anti-bovine TCRγδ (clone GB21A) diluted 1:100. Slides incubated with anti-TCRγδ antibody were washed and incubated for 30 min at room temperature with the polyclonal rabbit anti-mouse biotinylated secondary antibody (Dako) diluted 1:200 and then with the avidin–biotin peroxidase complex (Dako), for a further 30 min. The colour in all slides was developed by incubation with 3,3′-diaminobenzidine (Dako). After counterstaining sections with Mayer’s Haematoxylin (Merck), slides were mounted in Entellan (Merck). A positive reaction was indicated by the presence of brown cytoplasmic staining.

### Statistical analysis

Statistical significance of results was determined by non-parametric Friedman test with Dunn’s multiple comparisons test calculated with GraphPad Prism 7.0 c software. (*P ≤ 0.05; **P ≤ 0.01; ***P ≤ 0.001; ****P ≤ 0.0001). The data presented is from 5 pooled independent experiments with n = 2–3 animals as indicated in respective figure legends.

## Supplementary information

Supplementary Information

## Data Availability

The datasets generated during and/or analysed during the current study are available from the corresponding author on reasonable request.
