# Supplementary material for: T cells in mesenteric and subcutaneous adipose tissue of Holstein-Friesian cows
Source: Sci Rep. 2019 Mar 4;9:3413. doi: 10.1038/s41598-019-39938-0 (PMC6399275; doi:10.1038/s41598-019-39938-0)
Supplement: Supplementary file 1 — Supplementary Information [file 41598_2019_39938_MOESM1_ESM.pdf]

T cells in mesenteric and subcutaneous adipose tissue of Holstein-Friesian cows

Bárbara M. Oliveira[1,2], Ana M. Rasteiro[1,2], Alexandra Correia[3], Ana Pinto[1,2], Pedro Meireles[1,4], Paula G. Ferreira[1,2], Manuel Vilanova[1,3], Luzia Teixeira[1,2,*]

[1]ICBAS – Instituto de Ciências Biomédicas Abel Salazar, Universidade do Porto, Rua de Jorge Viterbo Ferreira, 228, 4050-313, Porto, Portugal.

[2]UMIB –Unidade Multidisciplinar de Investigação Biomédica, Universidade do Porto, Rua de Jorge Viterbo Ferreira, 228, 4050-313, Porto, Portugal.

[3]I3S-Instituto de Investigação e Inovação em Saúde, Universidade do Porto, Rua Alfredo Allen, 208, 4200-135 Porto, Portugal; IBMC – Instituto de Biologia Molecular e Celular, Rua Alfredo Allen, 208, 4200-135 Porto, Portugal.

[4] SVAExpleite, Rua D. Sancho I, 3202, 4760-485, Vila Nova de Famalicão, Portugal.

*Address Correspondence to:

Luzia Teixeira, PhD

ICBAS - Instituto de Ciências Biomédicas Abel Salazar da Universidade do Porto. Rua de Jorge Viterbo Ferreira n.º 228, 4050-313 Porto, Portugal.

Telephone: (+351) 220428109; E-mail: lmteixeira@icbas.up.pt

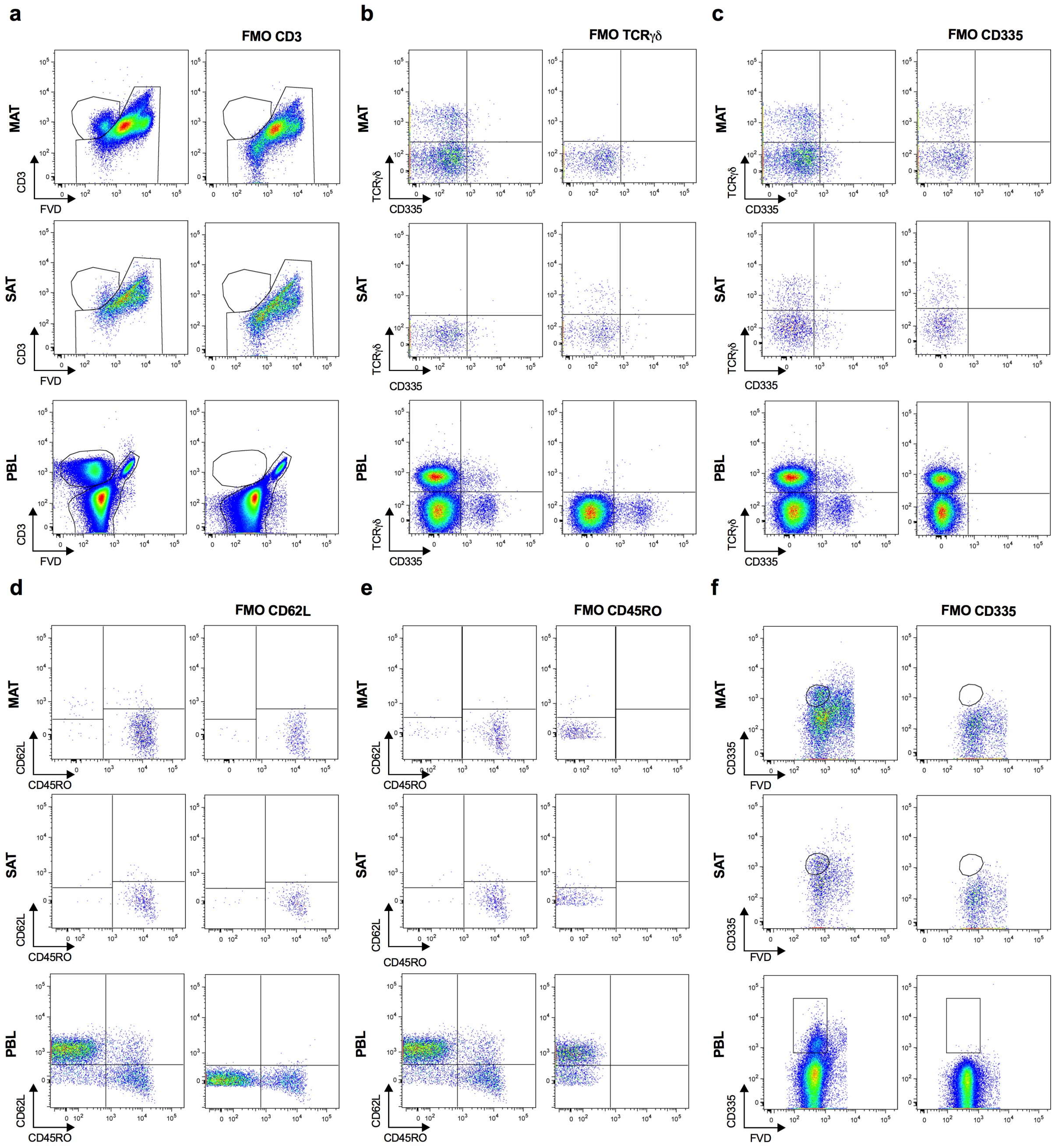

Supplementary Figure S1. Fluorescence Minus One controls for gating strategy. Representative pseudocolor plots of stained cells and respective Fluorescence Minus One (FMO) controls used to determine correct gating for T cells subpopulations. **(a)** Gate used to select CD3$^+$ cells (left plot) and respective FMO (right plot), **(b)** gate used to select TCRγδ$^+$ cells (left plot) and respective FMO (right plot), **(c)** gate used to selected CD335$^-$ cells and respective FMO (right plot), **(d)** gate used to select CD62L$^+$ cells in CD8$^+$ T cells (left plot) and respective FMO (right plot), **(e)** gate used to select CD45RO$^+$ cells in CD8$^+$ cells (left plot) and respective FMO (right plot), **(f)** gate used to select CD335$^+$ cells in CD3$^-$ cells (left plot) and respective FMO (right plot) in bovine mesenteric adipose tissue (MAT), subcutaneous adipose tissue (SAT) and peripheral blood leukocytes (PBL).

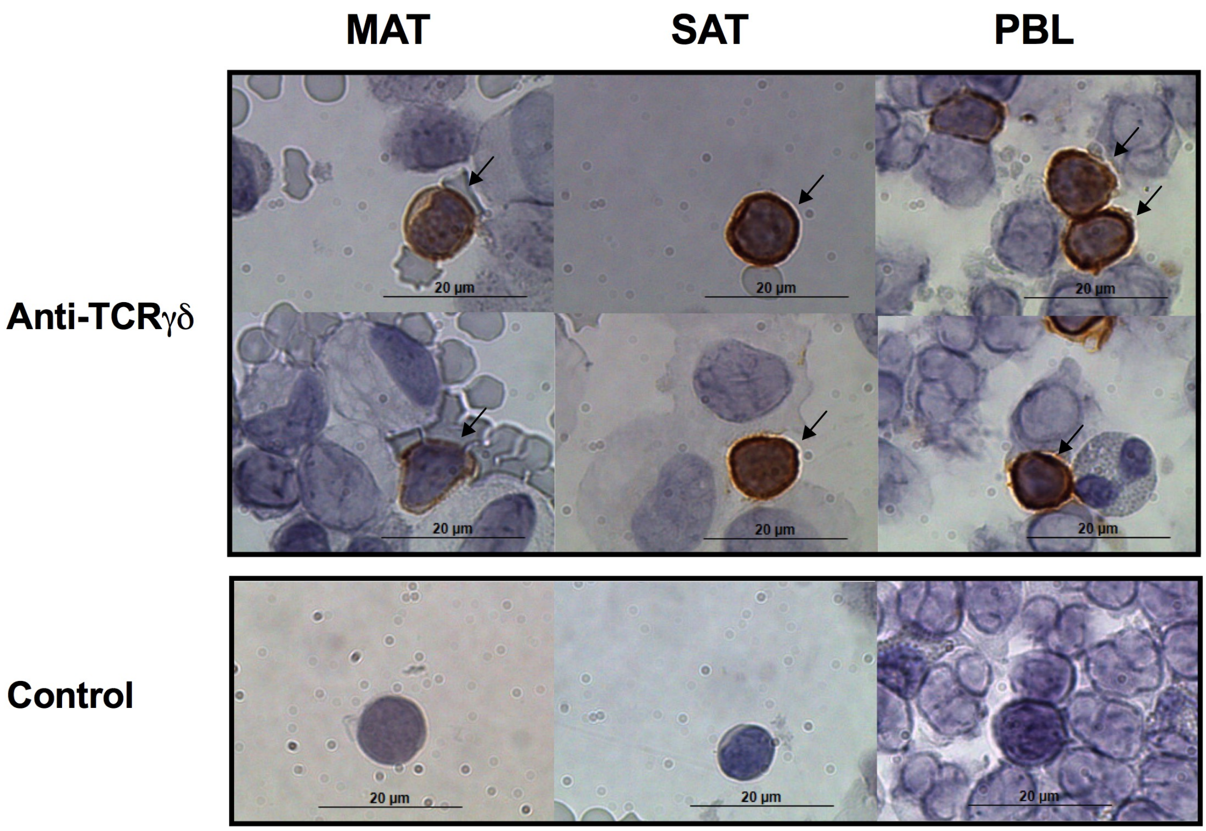

Supplementary Figure S2. Immunocytochemistry analysis of TCRγδ cells. Immunocytochemistry analysis of TCRγδ in stromal vascular fraction cells isolated from mesenteric and subcutaneous bovine adipose tissue (MAT and SAT, respectively) and in peripheral blood leukocytes (PBL). Cells were specifically stained (brown coloration, indicated by arrows) with a monoclonal mouse anti-bovine TCRγδ and counterstained with haematoxylin. Control (cells without addition of TCRγδ antibody). Bar=20µm.

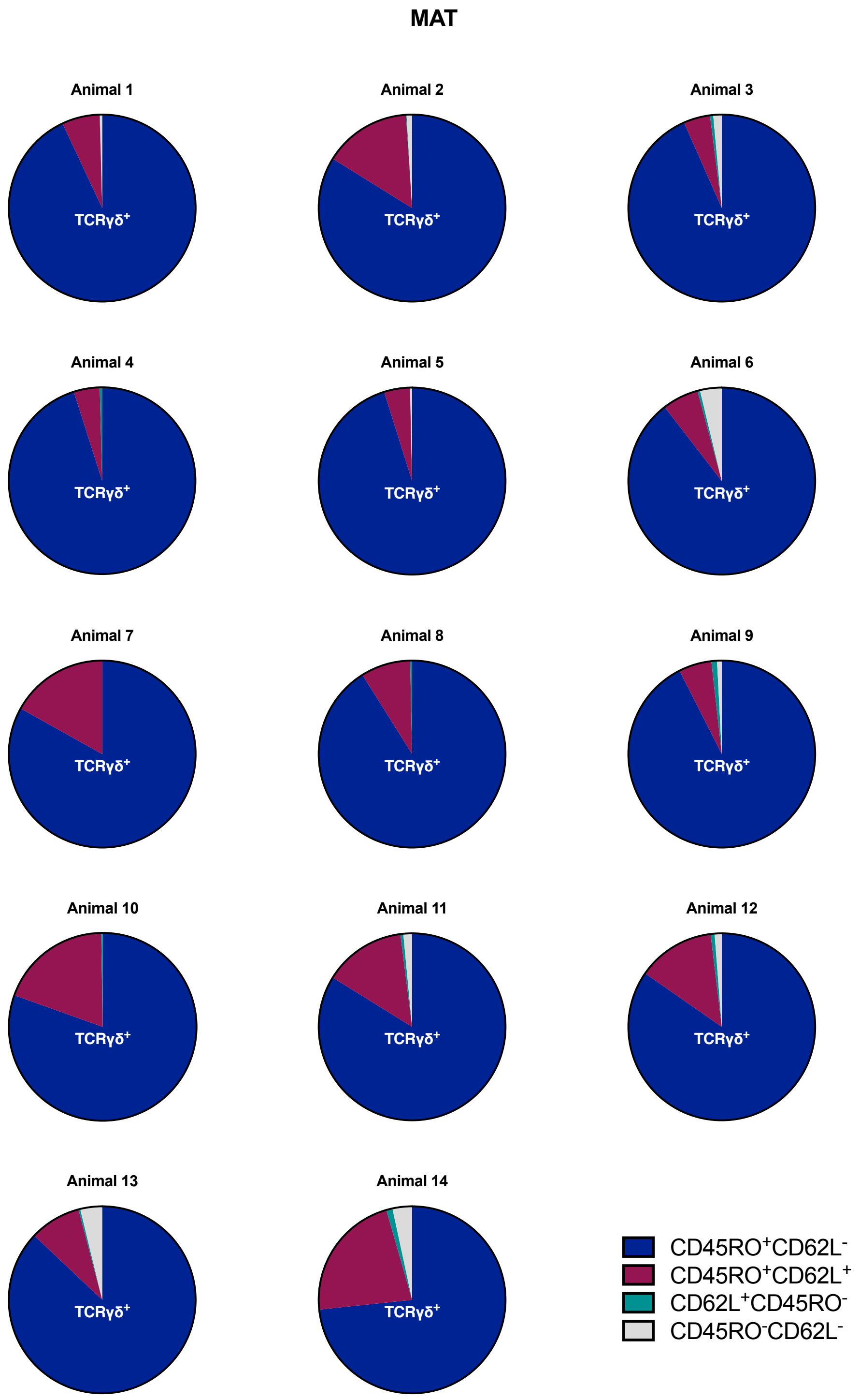

Supplementary Figure S3. Individual frequencies of CD45RO^{+} and CD62L^{+} cells on total γδ T cells in bovine mesenteric adipose tissue. Individual pie charts showing the relative frequency of CD45RO^{+}CD62L^{-} (blue), CD45RO^{+}CD62L^{+} (red), CD62L^{+}CD45RO^{-} (green) and CD45RO^{-}CD62L^{-} (grey) cells on total γδ T cells (TCRγδ^{+}CD3^{+}CD335^{-}) in mesenteric adipose tissue (MAT) from each animal included in the study.

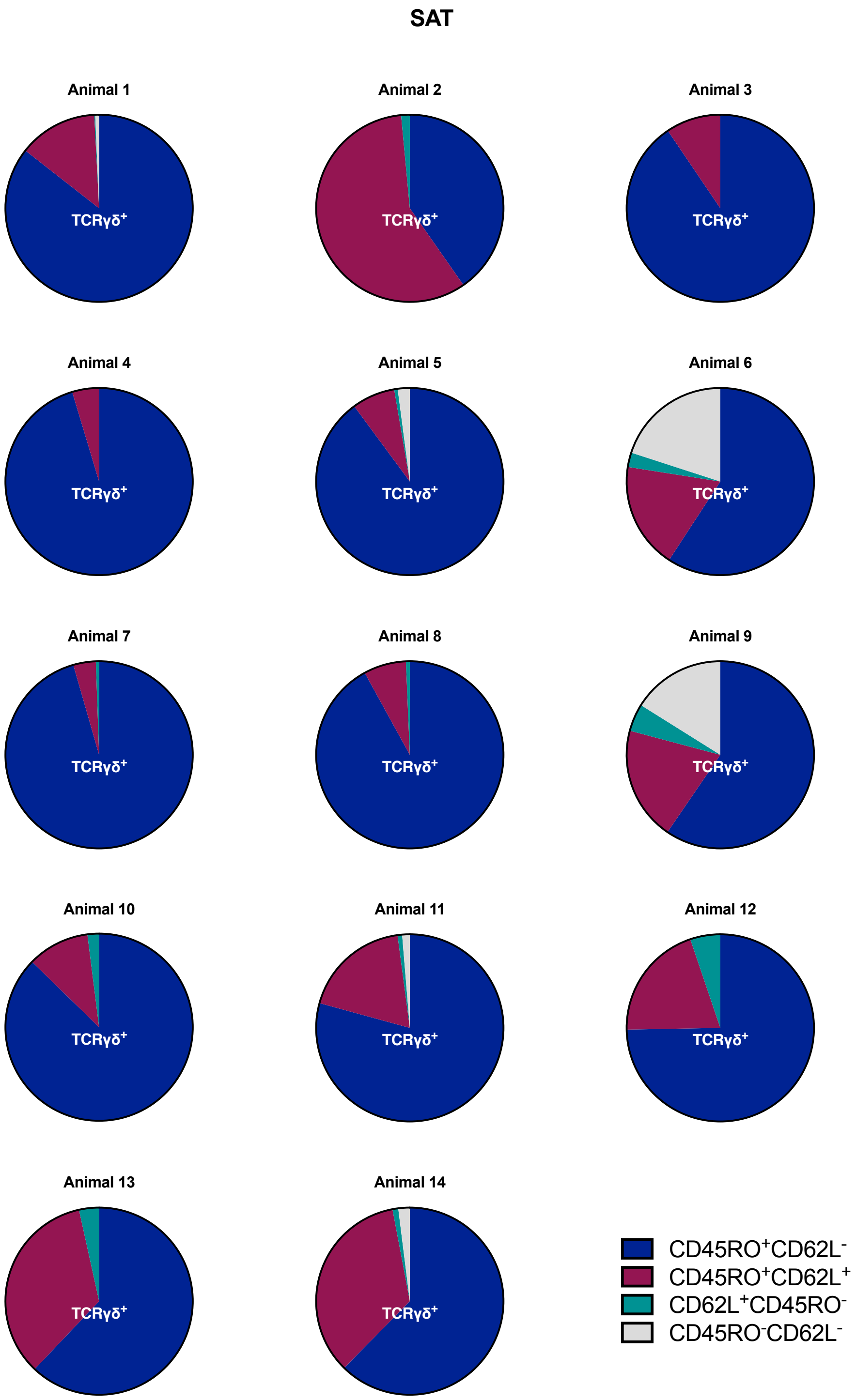

Supplementary Figure S4. Individual frequencies of CD45RO$^+$ and CD62L$^+$ cells on total γδ T cells in bovine subcutaneous bovine adipose tissue. Individual pie charts showing the relative frequency of CD45RO$^+$CD62L$^-$ (blue), CD45RO$^+$CD62L$^+$ (red), CD62L$^+$CD45RO$^-$ (green) and CD45RO$^-$CD62L$^-$ (grey) cells on total γδ T cells (TCRγδ$^+$CD3$^+$CD335$^-$) in subcutaneous adipose tissue (SAT) from each animal included in the study.

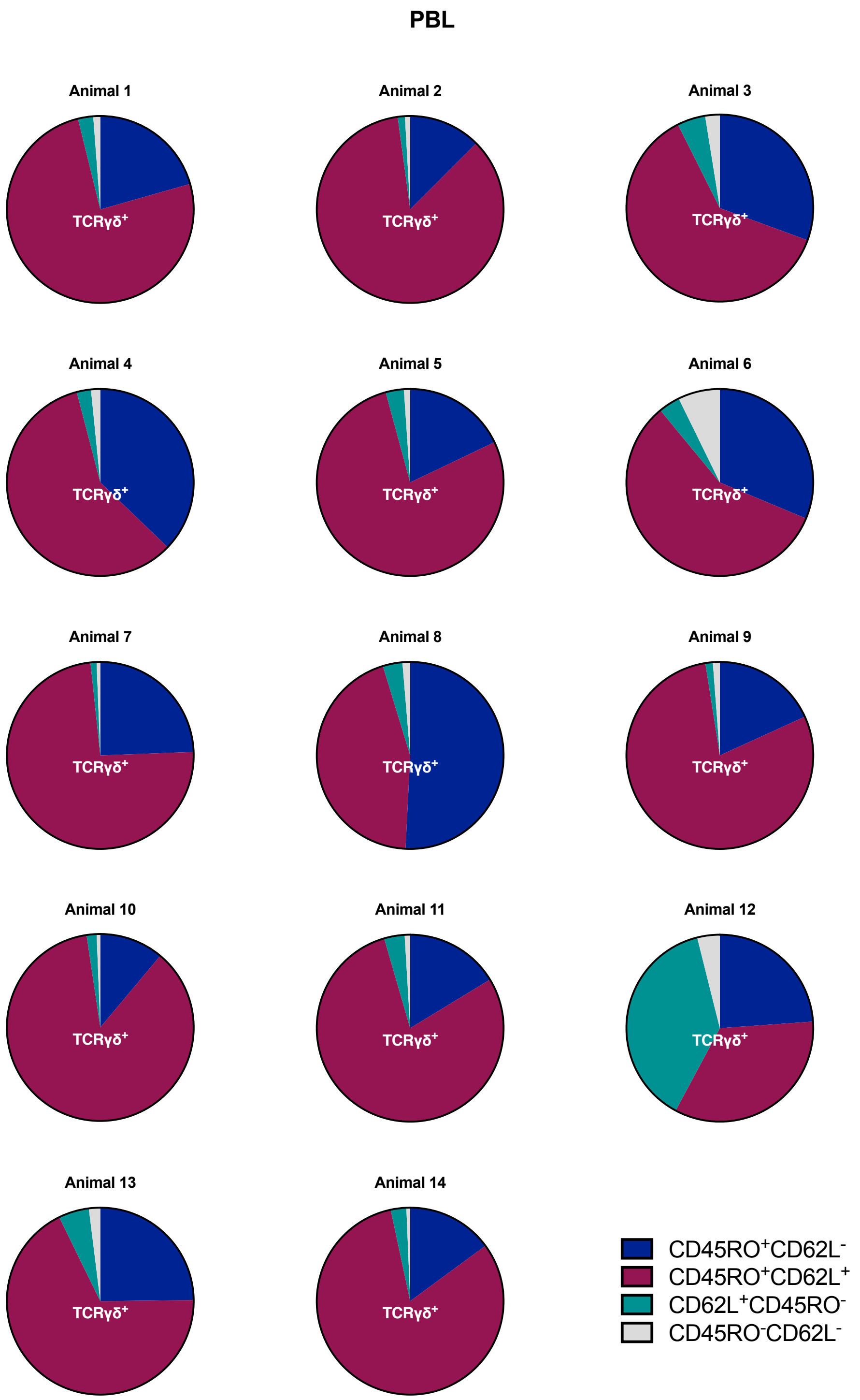

Supplementary Figure S5. Individual frequencies of CD45RO^{+} and CD62L^{+} cells on total γδ T cells in bovine peripheral blood leukocytes. Individual pie charts showing the relative frequency of CD45RO^{+}CD62L^{-} (blue), CD45RO^{+}CD62L^{+} (red), CD62L^{+}CD45RO^{-} (green) and CD45RO^{-}CD62L^{-} (grey) cells on total γδ T cells (TCRγδ^{+}CD3^{+}CD335^{-}) in peripheral blood leukocytes (PBL) from each animal included in the study.

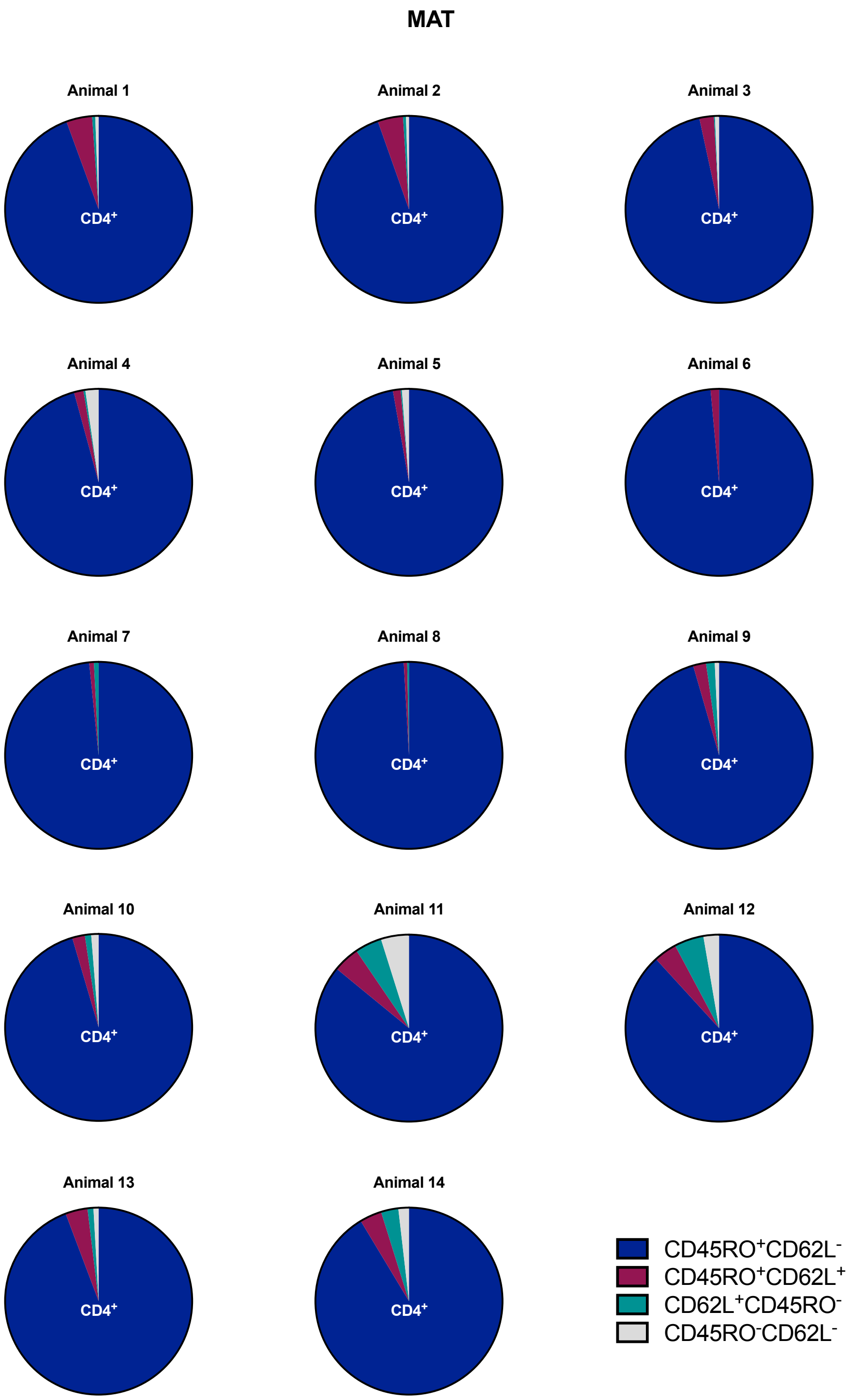

Supplementary Figure S6. Individual frequencies of CD45RO$^+$ and CD62L$^+$ cells on total CD4$^+$ T cells in bovine mesenteric adipose tissue. Individual pie charts showing the relative frequency of CD45RO$^+$CD62L$^-$ (blue), CD45RO$^+$CD62L$^+$ (red), CD62L$^+$CD45RO$^-$ (green) and CD45RO$^-$CD62L$^-$ (grey) cells on total CD4$^+$ T cells (CD4$^+$CD3$^+$TCRγδ$^-$CD335$^-$) in mesenteric adipose tissue (MAT) from each animal included in the study.

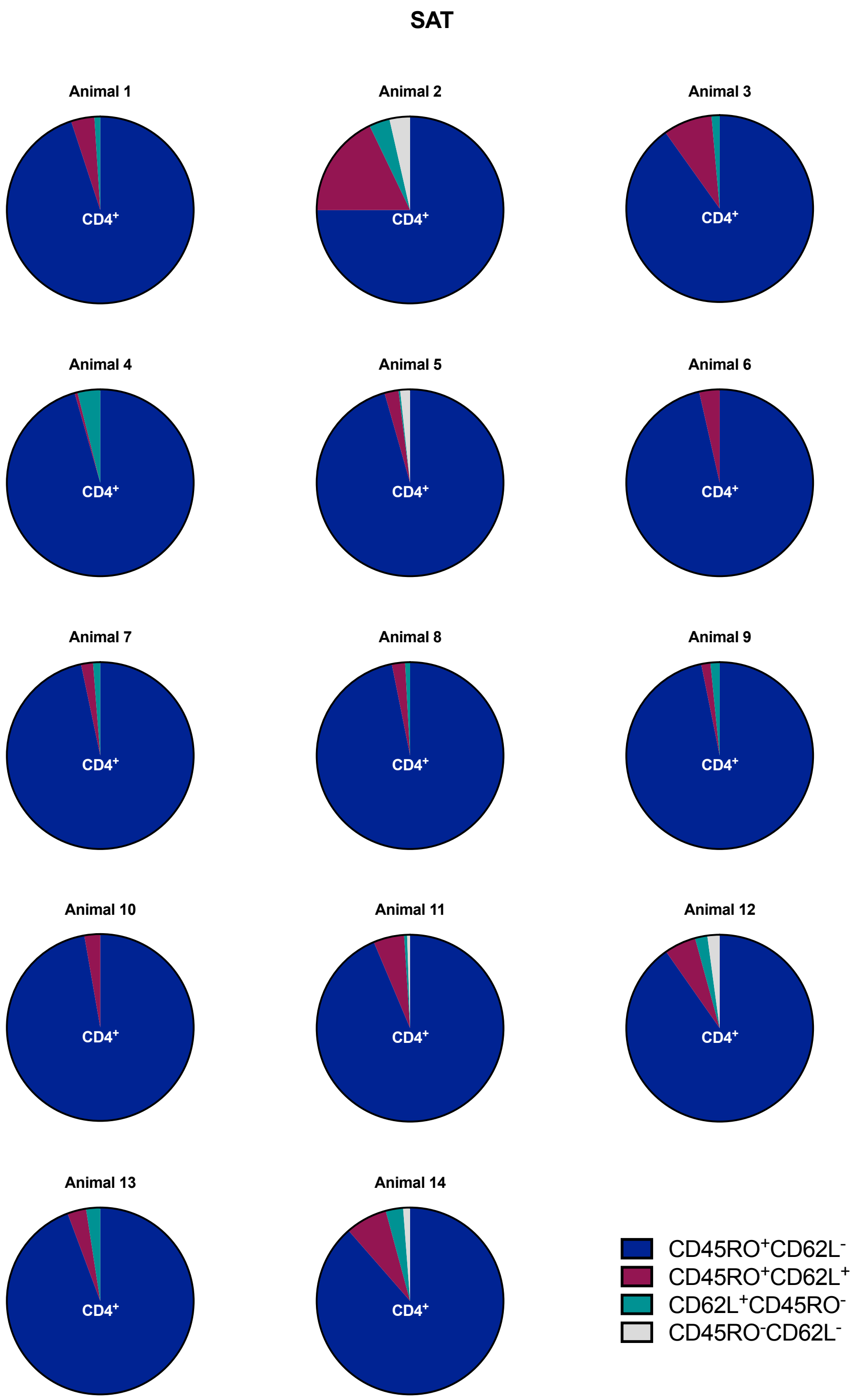

Supplementary Figure S7: Individual frequencies of CD45RO^{+} and CD62L^{+} cells on total CD4^{+} T cells in bovine subcutaneous adipose tissue. Individual pie charts showing the relative frequency of CD45RO^{+}CD62L^{-} (blue), CD45RO^{+}CD62L^{+} (red), CD62L^{+}CD45RO^{-} (green) and CD45RO^{-}CD62L^{-} (grey) cells on total CD4^{+} T cells (CD4^{+}CD3^{+}TCRγδ^{-}CD335^{-}) in subcutaneous adipose tissue (SAT) from each animal included in the study.

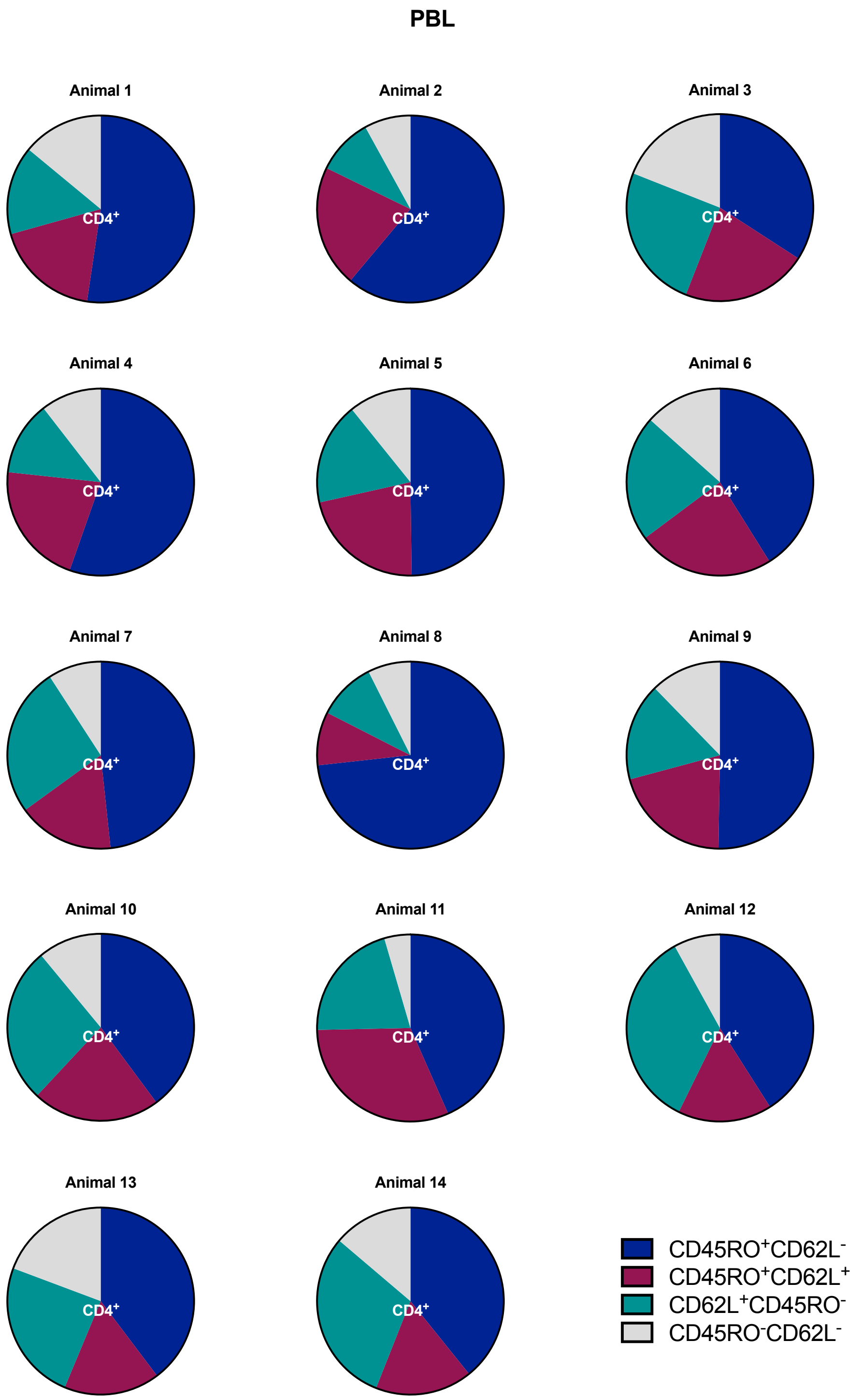

Supplementary Figure S8. Individual frequencies of CD45RO^{+} and CD62L^{+} cells on total CD4^{+} T cells in bovine peripheral blood leukocytes. Individual pie charts showing the relative frequency of CD45RO^{+}CD62L^{-} (blue), CD45RO^{+}CD62L^{+} (red), CD62L^{+}CD45RO^{-} (green) and CD45RO^{-}CD62L^{-} (grey) cells on total CD4^{+} T cells (CD4^{+}CD3^{+}TCR$\gamma\delta^{-}$CD335^{-}) in peripheral blood leukocytes (PBL) from each animal included in the study.

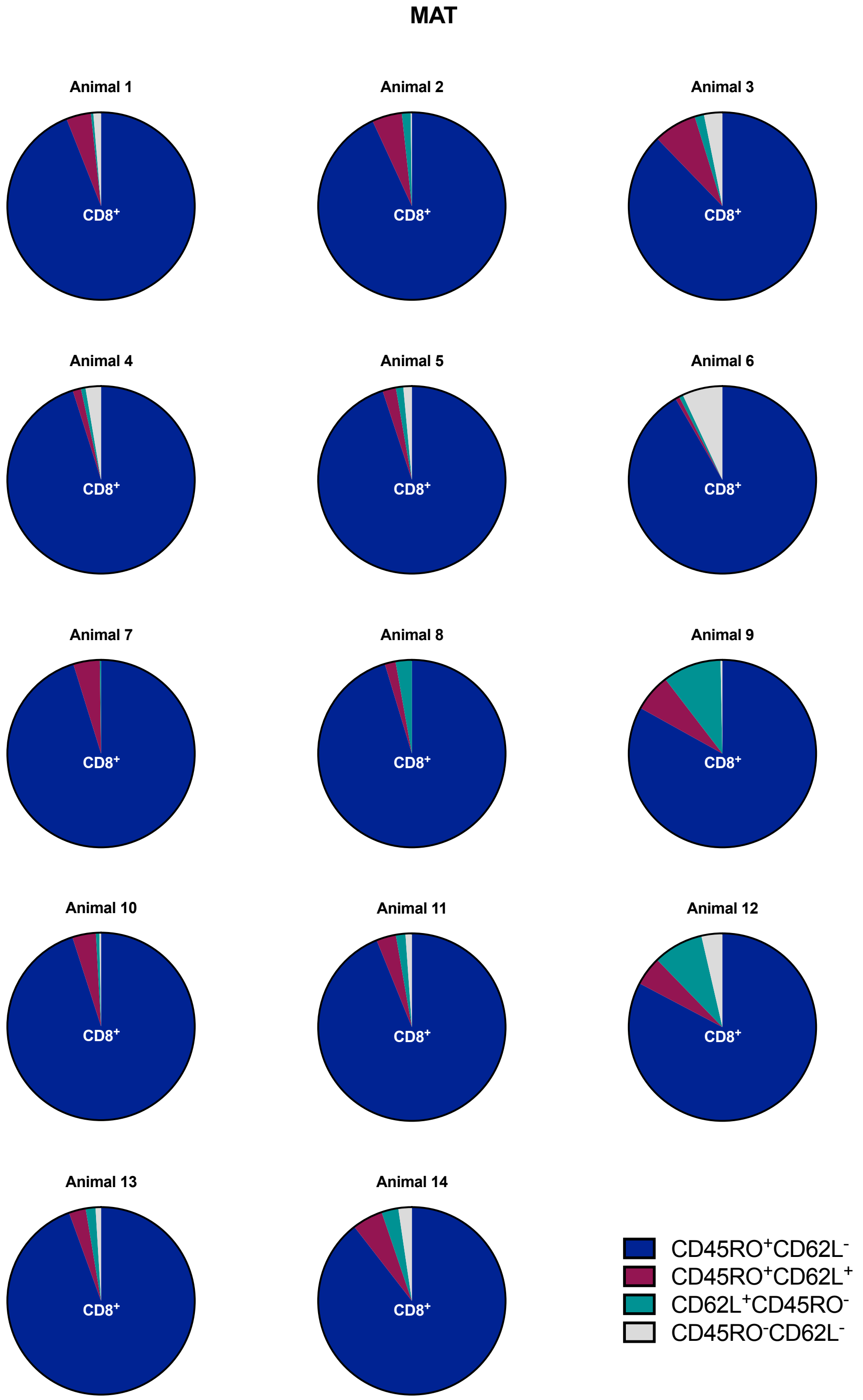

Supplementary Figure S9: Individual frequencies of CD45RO$^+$ and CD62L$^+$ cells on total CD8$^+$ T cells in bovine mesenteric adipose tissue. Individual pie charts showing the relative frequency of CD45RO$^+$CD62L$^-$ (blue), CD45RO$^+$CD62L$^+$ (red), CD62L$^+$CD45RO$^-$ (green) and CD45RO$^-$CD62L$^-$ (grey) cells on total CD8$^+$ T cells (CD8$^+$CD3$^+$TCRγδ$^-$CD335$^-$) in mesenteric adipose tissue (MAT) from each animal included in the study.

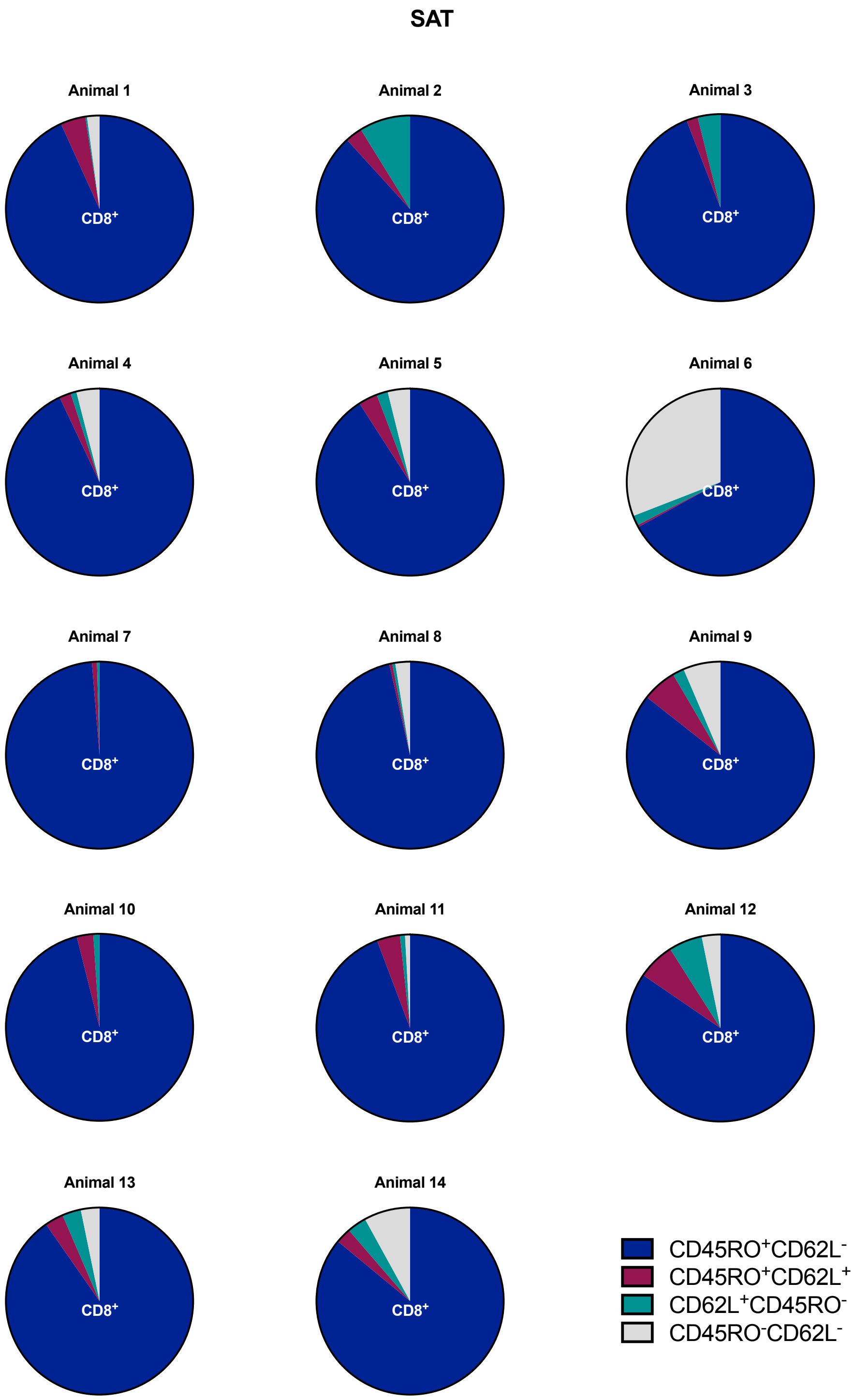

Supplementary Figure S10: Individual frequencies of $CD45RO^+$ and $CD62L^+$ cells on total $CD8^+$ T cells in bovine subcutaneous adipose tissue. Individual pie charts showing the relative frequency of $CD45RO^+CD62L^-$ (blue), $CD45RO^+CD62L^+$ (red), $CD62L^+CD45RO^-$ (green) and $CD45RO^-CD62L^-$ (grey) cells on total $CD8^+$ T cells ($CD8^+CD3^+TCR\gamma\delta^-CD335^-$) in subcutaneous adipose tissue (SAT) from each animal included in the study.

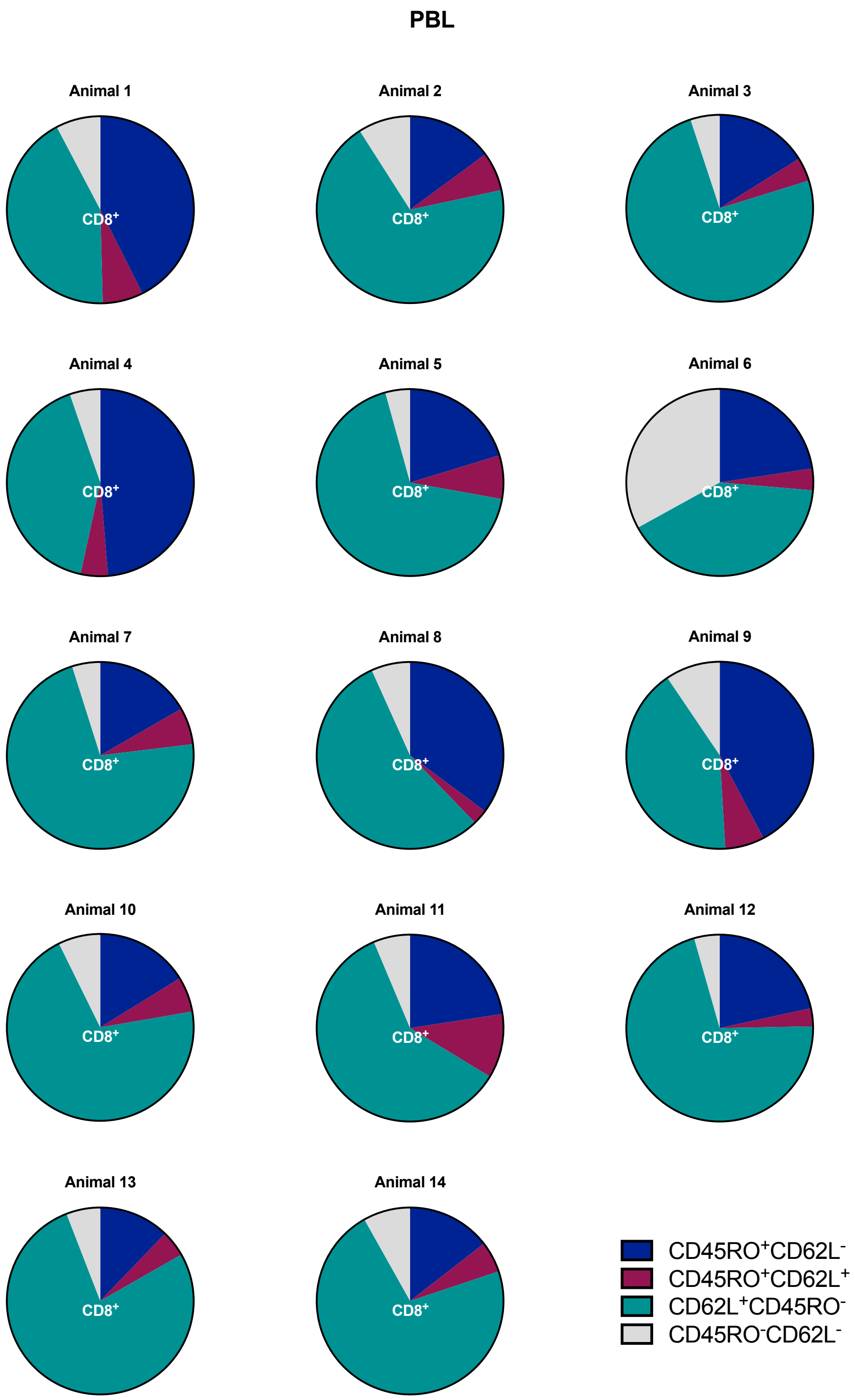

Supplementary Figure S11: Individual frequencies of $CD45RO^+$ and $CD62L^+$ cells on total $CD8^+$ T cells in bovine peripheral blood leukocytes. Individual pie charts showing the relative frequency of $CD45RO^+CD62L^-$ (blue), $CD45RO^+CD62L^+$ (red), $CD62L^+CD45RO^-$ (green) and $CD45RO^-CD62L^-$ (grey) cells on total $CD8^+$ T cells ($CD8^+CD3^+TCR\gamma\delta^-CD335^-$) in peripheral blood leukocytes (PBL) from each animal included in the study.

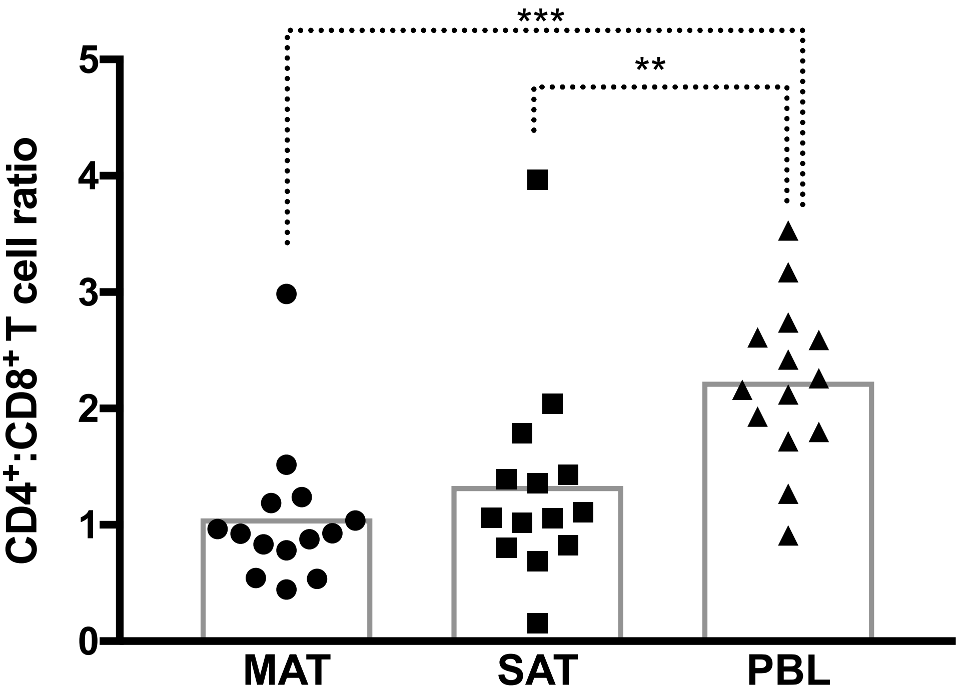

Supplementary Figure S12: $CD4^+$:$CD8^+$ T cell ratio. $CD4^+$:$CD8^+$ T cell ratio in mesenteric adipose tissue (MAT), subcutaneous adipose tissue (SAT) and peripheral blood leukocytes (PBL) obtained from 14 bovines pooled from 5 independent experiments is shown. Each symbol represents an individual animal. Bars represent means of 14 bovines per group pooled from 5 independent experiments. (Friedman test with Dunn's multiple comparisons test; **, P<0.01; ***, P<0.001).

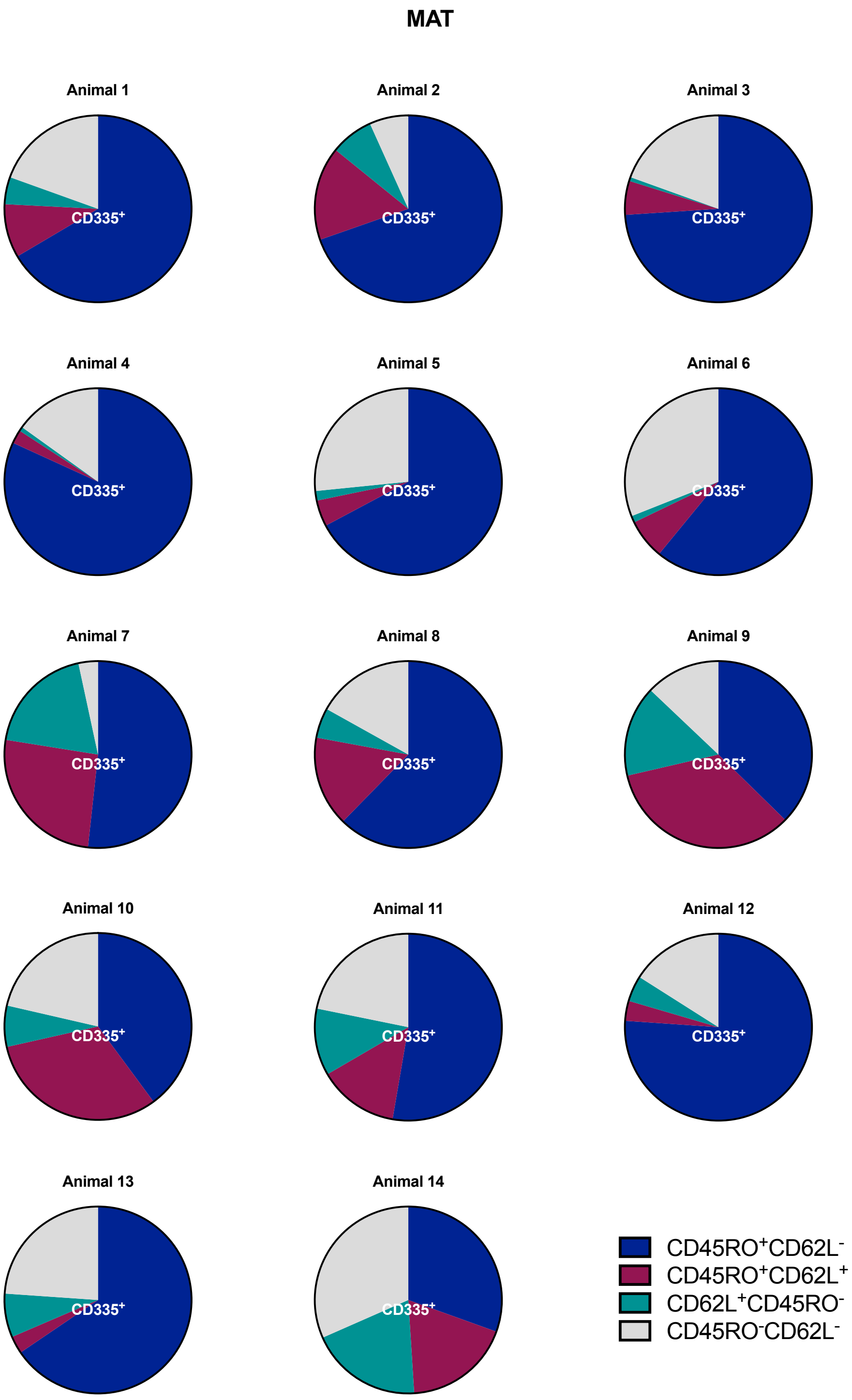

Supplementary Figure S13. Individual frequencies of $CD45RO^+$ and $CD62L^+$ cells on total NK cells in mesenteric bovine adipose tissue. Individual pie charts showing the relative frequency of $CD45RO^+CD62L^-$ (blue), $CD45RO^+CD62L^+$ (red), $CD62L^+CD45RO^-$ (green) and $CD45RO^-CD62L^-$ (grey) cells on total NK cells ($CD335^+CD3^-$) in mesenteric adipose tissue (MAT) from each animal included in the study.

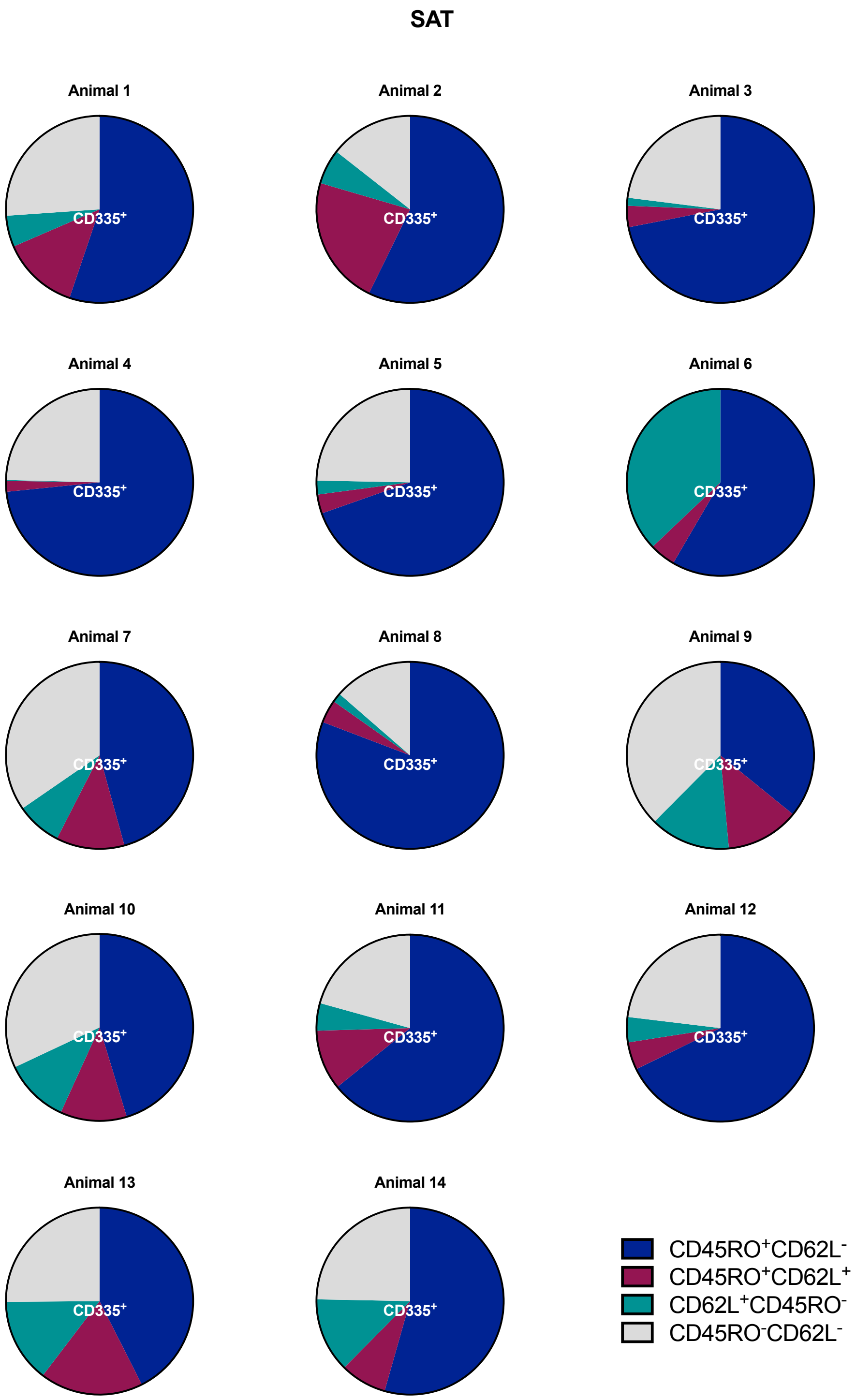

Supplementary Figure S14. Individual frequencies of $CD45RO^{+}$ and $CD62L^{+}$ cells on total NK cells in subcutaneous bovine adipose tissue. Individual pie charts showing the relative frequency of $CD45RO^{+}CD62L^{-}$ (blue), $CD45RO^{+}CD62L^{+}$ (red), $CD62L^{+}CD45RO^{-}$ (green) and $CD45RO^{-}CD62L^{-}$ (grey) cells on total NK cells ($CD335^{+}CD3^{-}$) in subcutaneous adipose tissue (SAT) from each animal included in the study.

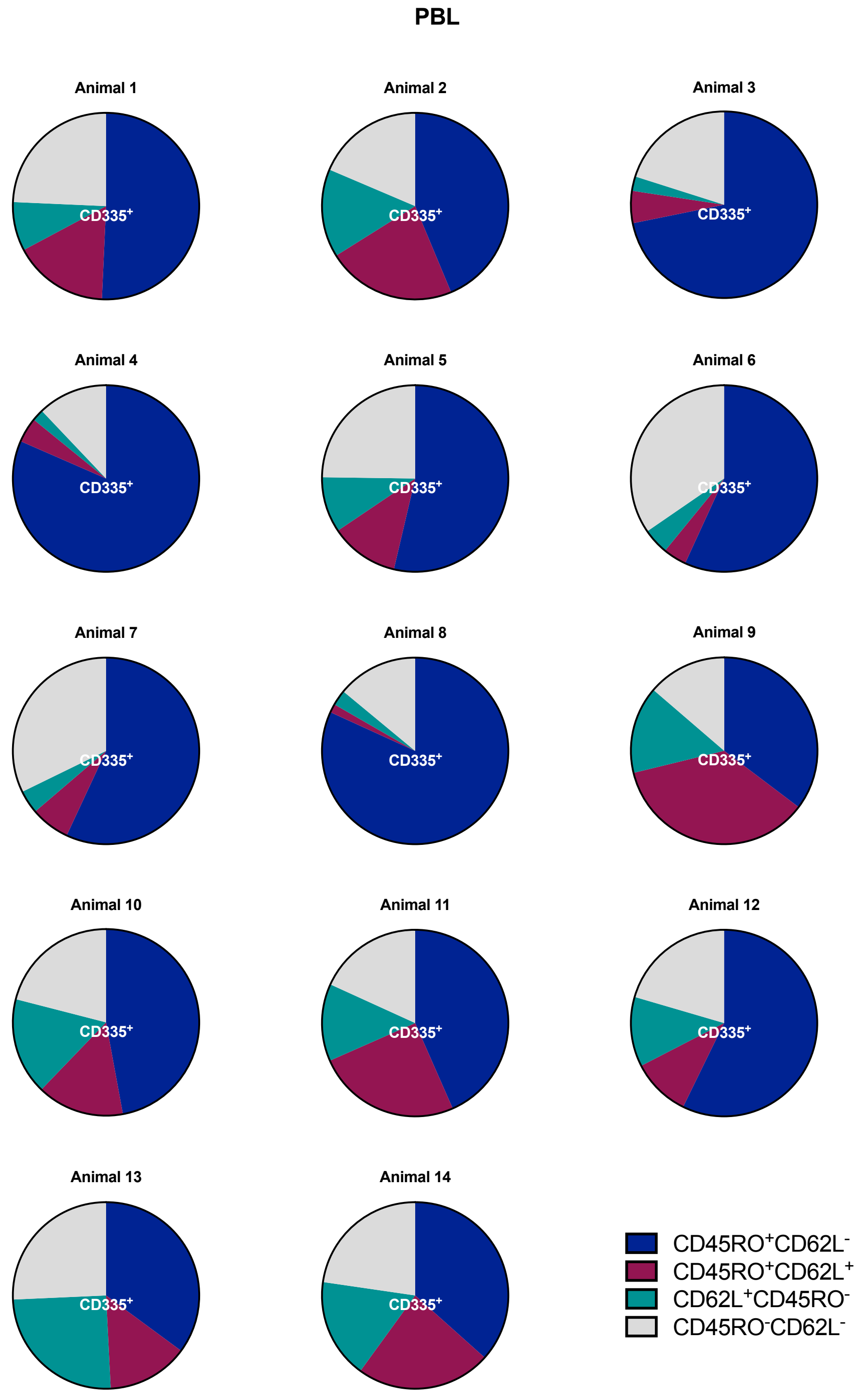

Supplementary Figure S15: Individual frequencies of $CD45RO^+$ and $CD62L^+$ cells on total NK cells in peripheral blood leukocytes. Individual pie charts showing the relative frequency of $CD45RO^+CD62L^-$ (blue), $CD45RO^+CD62L^+$ (red), $CD62L^+CD45RO^-$ (green) and $CD45RO^-CD62L^-$ (grey) cells on total NK cells ($CD335^+CD3^-$) in peripheral blood leukocytes (PBL) from each animal included in the study.

Supplementary Table S1. Age of the animals included in this study in months.

Animal number	Age (months)
**1**	9
**2**	11
**3**	66
**4**	101
**5**	49
**6**	97
**7**	25
**8**	49
**9**	20
**10**	24
**11**	27
**12**	39
**13**	59
**14**	22
